# Impact of Maternal Intake of Artificial Sweetener, Acesulfame-K, on Metabolic and Reproductive Health Outcomes in Male and Female Mouse Offspring

**DOI:** 10.3389/fnut.2021.745203

**Published:** 2021-12-06

**Authors:** Pania E. Bridge-Comer, Mark H. Vickers, Jacob Morton-Jones, Ana Spada, Jing Rong, Clare M. Reynolds

**Affiliations:** ^1^Liggins Institute, University of Auckland, Auckland, New Zealand; ^2^School of Public Health, Physiotherapy and Sports Science/Conway Institute/Institute of Food and Health/Diabetes Complications Research Centre, University College Dublin, Belfield, Ireland

**Keywords:** artificial sweetener, metabolic health, reproductive, maternal nutrition, adipose tissue

## Abstract

Guidelines advising pregnant women to avoid food and beverages with high fat and sugar have led to an increase in the consumption of “diet” options sweetened by artificial sweeteners (AS). Yet, there is limited information regarding the impact of AS intake during pregnancy on the long-term risk of cardiometabolic and reproductive complications in adult offspring. This study examined the influence of maternal acesulfame-K (Ace-K) and fructose consumption on metabolic and reproductive outcomes in offspring. Pregnant C57BL/6 mice received standard chow *ad-libitum* with either water (CD), fructose (Fr; 20% kcal intake), or AS (AS; 12.5 mM Ace-K) throughout pregnancy and lactation (*n* = 8/group). Postweaning offspring were maintained on a CD diet for the remainder of the experiment. Body weight, food intake, and water intake were measured weekly. Oral glucose tolerance tests (OGTT) were undertaken at 12 weeks, and the offspring were culled at week 14. Female, but not male, AS groups exhibited decreased glucose tolerance compared to Fr. There was an increase in gonadal fat adipocyte size in male offspring from AS and Fr groups compared to CD groups. In female offspring, adipocyte size was increased in the Fr group compared to the CD group. In female, but not male offspring, there was a trend toward increase in *Fasn* gene expression in AS group compared to the CD group. Maternal AS and Fr also negatively impacted upon female offspring estrus cycles and induced alterations to markers associated with ovulation. In summary, exposure to Ace-k *via* the maternal diet leads to impaired glucose tolerance and impacts adipocyte size in a sex-specific manner as well as significantly affecting estrus cycles and related gene markers in female offspring. This has implications in terms of providing tailored dietary advice for pregnant women and highlights the potential negative influence of artificial sweetener intake in the context of intergenerational impacts.

## Introduction

The consumption of artificial sweeteners (AS) has increased in recent decades (1, 2), with beverages being a common mode of intake. This increase is partly due to the rising awareness regarding the link between sugar intake and the risk for obesity and other associated non-communicable diseases. However, despite the promoted beneficial effects of AS, a number of studies have suggested that AS consumption can elevate the risk of insulin resistance (IR), type 2 diabetes mellitus (T2DM), obesity, and cardiovascular disease (3–6), and negatively impact the reproductive system (7, 8). It is now well established that suboptimal conditions during pregnancy and lactation, including poor maternal nutrition, can have an impact on the risk for development of disorders in offspring, including cardiometabolic disease, obesity (9), and reproductive disorders such as polycystic ovarian syndrome (PCOS) or anovulation in later life (10, 11). In this context, AS consumption may influence the health of offspring due to negative impacts on development *in utero* and during the early neonatal period, although there remains a paucity of data in this area.

Pregnant women are frequent consumers of AS, with over a quarter (29.5%) of pregnant women reported consuming AS during pregnancy, of which over 5% do so daily (12, 13). In women diagnosed with gestational diabetes mellitus (GDM), just under half (45.4%) report AS intake during pregnancy, and 9.2% report daily intake (14). AS intake during pregnancy increases the risk of preterm birth as observed in both human and mouse studies (13, 15), while AS such as saccharin, sucralose, and acesulfame-K (Ace-K) have been found in breast milk samples from women nursing infants, indicating that these chemicals may then be passed on to the developing infant during lactation (16, 17). Evidence from human cohorts also implicates maternal AS consumption in the increased incidence of obesity in offspring at one (12), and 7 years of age (14). Further studies in mice have shown the presence of Ace-K in the amniotic fluid and the mother's milk following intraoral feeding, which directly increases male offspring's sweet-taste preference for Ace-K in adulthood (18). Previous work from this group in mice has also demonstrated that maternal AS and fructose (Fr) intake induces maternal metabolic dysfunction and negatively alters fetal development in mice (15).

However, despite growing evidence that suggests adverse impacts of AS consumption, the regulations and guidelines relating to AS intake, in particular during pregnancy, remain unclear (19). The Academy of Nutrition and Dietetics, for instance, deems consumption of AS during pregnancy acceptable (20). Contradictory evidence from studies indicating no significant metabolic impairments following consumption (21) confounds confusion around the safety of AS, particularly during pregnancy and lactation. Further, differences between types of AS highlight the need for comprehensive studies into those AS most prevalent in our diets, such as Ace-K, which is one of the most popular AS (22). In human studies, differences in findings may be due to various confounding factors such as interactions with other dietary intakes and the overall dietary pattern which makes delineation of the direct effects of AS difficult. Animal models are therefore a key tool for understanding the potential effects of AS intake due to the ability to more tightly control potential experimental confounders. However, there remains a paucity of information regarding the effects of a maternal diet of AS in relation to the metabolic and reproductive health of offspring in experimental animal models, despite the growing need for such information. The aim of this study was therefore to determine the influence of the AS, Ace-K, on metabolic markers in male and female offspring, and estrus cyclicity and related ovarian gene expression in females following maternal dietary exposures during pregnancy and lactation.

## Methods

### Animal Procedures

All animal procedures were approved by the Animal Ethics Committee at the University of Auckland (Approval number 001846) in accordance with the New Zealand Animal Welfare Act, 1999. Breeding pairs of C57BL/6 mice were purchased from the Vernon Jansen Unit at the University of Auckland and housed under standard conditions (wood shavings as bedding, 22°C, 40–45% humidity, and a 12-h light−12-h dark cycle).

All mice were maintained on a standard chow diet (Envigo, 2018 Teklad Global 18% Protein Rodent Diet, Ind, USA) *ad-libitum* throughout the experiment. Age-matched female mice were housed with an unrelated C57BL/6 male for mating at 10 weeks of age. Confirmation of pregnancy by vaginal plug was recorded as gestational day (GD) 0.5. Pregnant dams were then randomly assigned onto one of the following three dietary groups (*n* = 8/group):

- Control (CD; standard diet and drinking water)
- Artificial sweetener (AS; standard diet and 12.5 mM Ace-K in drinking water)
- Fructose (Fr; standard diet and 34.7 mM fructose in drinking water)

Artificial sweetener and Fr doses were calculated to be equivalent to a human dose of one standard can of soda a day. Diets were maintained throughout pregnancy and lactation. Date of birth was noted and marked as postnatal day 1 (P1). At postnatal day 2 (P2), litters were weighed, sex was determined using anogenital distance, and litters were randomly reduced to eight pups per litter (four males and four females) to standardize nutrition until weaning. Offspring were weaned at postnatal day 21 and one male and one female from each litter were assessed to represent true biological replicates across the eight litters generated per dietary group. Food and water intake were also measured weekly. All offspring were fed the standard control diet and water *ad-libitum* from weaning until the end of the experiment (14 weeks). An oral glucose tolerance test (OGTT) was carried out 2 weeks prior to cull. At 14 weeks of age, mice were fasted for 6 h and tail blood and plasma samples were collected as detailed below. Mice were then weighed and culled by cervical dislocation. Female mice were staged prior to cull to ensure all female offspring were in diestrus. Gonadal adipose tissue and, in female offspring, ovaries were dissected, weighed, and snap-frozen, subcutaneous fat was collected and snap-frozen, and then stored at −80°C for later analysis.

### Measurement of Pubertal Onset

Onset of puberty in female offspring was determined by sighting of vaginal canalization. Screening for puberty onset began at day 25, occurring every morning at a consistent time (0900h), and continued until vaginal canalization was observed. The age and body weights of offspring at the time of puberty onset were recorded.

### Estrus Staging

Estrus cycle staging was carried out through vaginal smear cytology, as detailed previously (23). Smearing began 5 weeks prior to cull to ensure a minimum of 5–6 cycles per animal. Smearing occurred at the same time every morning (0900h), using soft-tipped cotton buds dipped in clean saline solution (0.9%). Slides were stained by hematoxylin baths and the relative abundance of leukocytes, nucleated vaginal epithelial cells, and cornified epithelial cells in each smear were examined using light microscopy. Cycles were deemed regular if they exhibited the typical 4-day cycle, and irregular if a stage was extended or did not follow the regular pattern. Persistent estrus was determined by two or more days of estrus over one or more cycles.

### Oral Glucose Tolerance Test

At 12 weeks of age, mice were fasted for 6 h from 8 a.m. Following weighing, the tip of the tail was snipped (<1 mm) and the second drop of blood was read using a glucometer (Accu-Chek Performa, Roche Diabetes Care, Ind, USA). Mice received 2 g/kg D-glucose *via* oral gavage. Blood glucose concentrations were measured from the tail tip at 0, 15, 30, 60, 90, and 120 min. Tail blood was also collected into EDTA microvettes (CB300, Sarstedt, NC, USA) at 0, 15, and 60 min. Samples were centrifuged for 10 min/1500 x *g*/4°C, and the resulting plasma was stored at −20°C for plasma insulin analysis.

### Histological Analysis

Gonadal and subcutaneous adipose tissues were fixed in 10% neutral-buffered formalin, then paraffin embedded and sectioned (10 μm) using a Leica R 2,135 rotary microtome (Leica Instruments, Singapore). The slides underwent H&E staining and were then mounted with DPX mountant. Slides were visualized at 20x and images were captured using a light microscope and NIS Elements-D software (Nikon 800). Four representative images of each section were taken and analyzed in a blinded manner with ImageJ software (NIH) to determine the mean adipocyte size and distribution.

### Plasma Analysis

The mouse-specific insulin, leptin, and testosterone ELISA kits (Ultra Sensitive Mouse Insulin ELISA Kit (Cat. # 90080), the Mouse Leptin ELISA kit (Cat. # 90030), and the Mouse Testosterone ELISA Kit (Cat. # 80552), Crystal Chem Inc., IL, USA) were used according to the manufacturer's instructions.

### Gene Expression Analysis

Female ovarian RNA was extracted using RNeasy Mini Kits (Cat. No 74104, Qiagen, Hilden, Germany) and a TissueLyser (Qiagen, Hilden, Germany) as per the manufacturers' instructions.

Adipose tissue RNA was extracted using the trizol extraction method and a TissueLyser (Qiagen, Hilden, Germany). Following homogenization, the lysate was centrifuged for 10 min/1200 x *g*/4°C, and the accumulated fat layer was removed to improve RNA yield. Following the addition of isopropanol, samples were incubated overnight at −20°C.

Following extraction, all RNA were assessed with a NanoDrop spectrophotometer (NanoPhotometer N60, Implen). A High Capacity cDNA Reverse Transcription Kit (Life Technologies Ltd., Applied Biosystems, MS, USA) was used to generate cDNA as per the manufacturer's instructions. Polymerase chain reaction (PCR) was performed using the Applied Biosystems QuantStudio 6 Flex Real-Time PCR system (Applied Biosystems). Genes were normalized to the geomean of *Rps29* and *Rps13* expression. The comparative CT method was utilized to analyze the results (24).

### Statistical Analysis

Statistical analysis was performed using GraphPad Prism and IBM SPSS Statistics Data Editor Version 27. The Shapiro–Wilk test was used to assess normality. Any data that was not normally distributed were transformed as appropriate. Female and male offspring data were analyzed separately. Repeated measures two-way ANOVA was performed for the OGTT data. Estrus cyclicity data were analyzed using nominal logistic regression. All other data were analyzed using the one-way ANOVA. The Holm–Sidak *post-hoc* tests were performed as indicated for comparison testing between the groups. Significance between the groups was considered at *P* < 0.05. Unless otherwise stated, all data are presented as mean ± SEM with an *n* = 8 per sex per maternal dietary group.

## Results

### Body Weight and Caloric Intake

Maternal AS and Fr consumption had no impact on offspring birth, weaning, or final cull weight in either female or male offspring. Similarly, there was no difference in absolute gonadal fat weight or when expressed relative to body weight in either female or male offspring. There was no effect of maternal dietary group on ovary or testes weights. There was no difference between groups in relation to male/female ratios and litter size (Table 1).

**Table 1:** Birth, weaning, and cull weight, and gonads and gonadal fat weight of female and male offspring.

	**CD**	**AS**	**Fr**	**Main effect (*P* < 0.05)**
**Females**				
Birth weight (g)	1.38 ± 0.06	1.30 ± 0.03	1.36 ± 0.02	NS
Weaning weight (g)	8.06 ± 0.62	7.86 ± 0.56	7.74 ± 0.71	NS
Cull weight (g)	20.07 ± 0.39	19.52 ± 0.27	20.21 ± 0.35	NS
Ovaries (g)	0.012 ± 0.003	0.013 ± 0.003	0.013 ± 0.003	NS
Ovaries (% BW)	0.060 ± 0.003	0.061 ± 0.004	0.059 ± 0.003	NS
Gonadal fat (g)	0.32 ± 0.13	0.30 ± 0.14	0.31 ± 0.11	NS
Gonadal fat (% BW)	1.48 ± 0.14	1.37 ± 0.14	1.45 ± 0.11	NS
Pubertal onset (day)	30.67 ± 0.54	31.35 ± 0.71	32.25 ± 0.64	NS
Weight at pubertal onset (g)	14.42 ± 0.3	14.57 ± 0.3	15.12 ± 0.4	NS
**Males**				
Birth weight (g)	1.43 ± 0.05	1.40 ± 0.03	1.36 ± 0.03	NS
Weaning weight (g)	8.30 ± 0.26	8.23 ± 0.42	7.65 ± 0.26	NS
Cull weight (g)	26.28 ± 0.45	26.01 ± 0.26	25.57 ± 0.75	NS
Testes (g)	0.21 ± 0.014	0.21 ± 0.023	0.21 ± 0.011	NS
Testes (% BW)	0.77 ± 0.014	0.78 ± 0.019	0.77 ± 0.017	NS
Gonadal fat (g)	0.44 ± 0.17	0.46 ± 0.15	0.43 ± 0.12	NS
Gonadal fat (% BW)	1.55 ± 0.14	1.71 ± 0.13	1.55 ± 0.11	NS
Male/Female Ratio	0.91 ± 0.16	1.18 ± 0.23	1.02 ± 0.19	NS
Litter size	7.88 ± 0.48	8.38 ± 0.46	8.25 ± 0.25	NS

*Puberty onset and weight at pubertal onset of female offspring, male/female ratio, and litter size. Data presented as mean ± SEM. Birth and weaning weights represent eight litters per group. For all other measures n = 8 per sex per group. NS, Not significant*.

### Plasma Analysis

Plasma leptin and insulin concentrations and HOMA-IR index were unchanged by either maternal AS or Fr consumption in female and male mice. There was no change in fasting plasma glucose concentrations at cull in female offspring. However, in male mice, AS reduced glucose concentrations compared to both CD and Fr groups. Maternal diet had no effect on the testosterone concentrations in female offspring. However, in male mice, maternal Fr intake significantly increased the plasma testosterone concentrations compared to CD and AS groups (Table 2).

**Table 2:** Plasma biochemical analysis in female and male offspring.

	**CD**	**AS**	**Fr**	**Main effect (*P* < 0.05)**
**Females**				
Leptin (ng/ml)	1.49 ± 0.20	1.56 ± 0.25	1.63 ± 0.20	NS
Insulin (ng/ml)	0.47 ± 0.049	0.39 ± 0.041	0.43 ± 0.044	NS
Glucose (mmol/l)	8.82 ± 0.22	8.44 ± 0.18	8.34 ± 0.29	NS
HOMA-IR	1.89 ± 0.18	1.52 ± 0.17	1.63 ± 0.17	NS
Testosterone (ng/ml)	0.37 ± 0.074	0.51 ± 0.099	0.47 ± 0.094	NS
**Males**				
Leptin (ng/ml)	1.57 ± 0.48	1.22 ± 0.20	0.85 ± 0.18	NS
Insulin (ng/ml)	0.75 ± 0.064	0.79 ± 0.065	0.81 ± 0.078	NS
Glucose (mmol/l)	9.22 ± 0.19	8.35 ± 0.22^*^	9.49 ± 0.25^+^	0.002
HOMA-IR	3.18 ± 0.33	3.12 ± 0.27	3.53 ± 0.32	NS
Testosterone (ng/ml)	1.92 ± 0.83	0.90 ± 0.29	8.92 ± 2.67^*+^	0.002

Data are presented as mean ± SEM, where ^*^p < 0.05 w.r.t CD, +p < 0.05 w.r.t AS; n = 8 per sex per group. NS, Not significant. HOMA, fasting insulin (microU/L) × fasting glucose (nmol/L)/22.5.

### Oral Glucose Tolerance Tests

**Figure 1:**
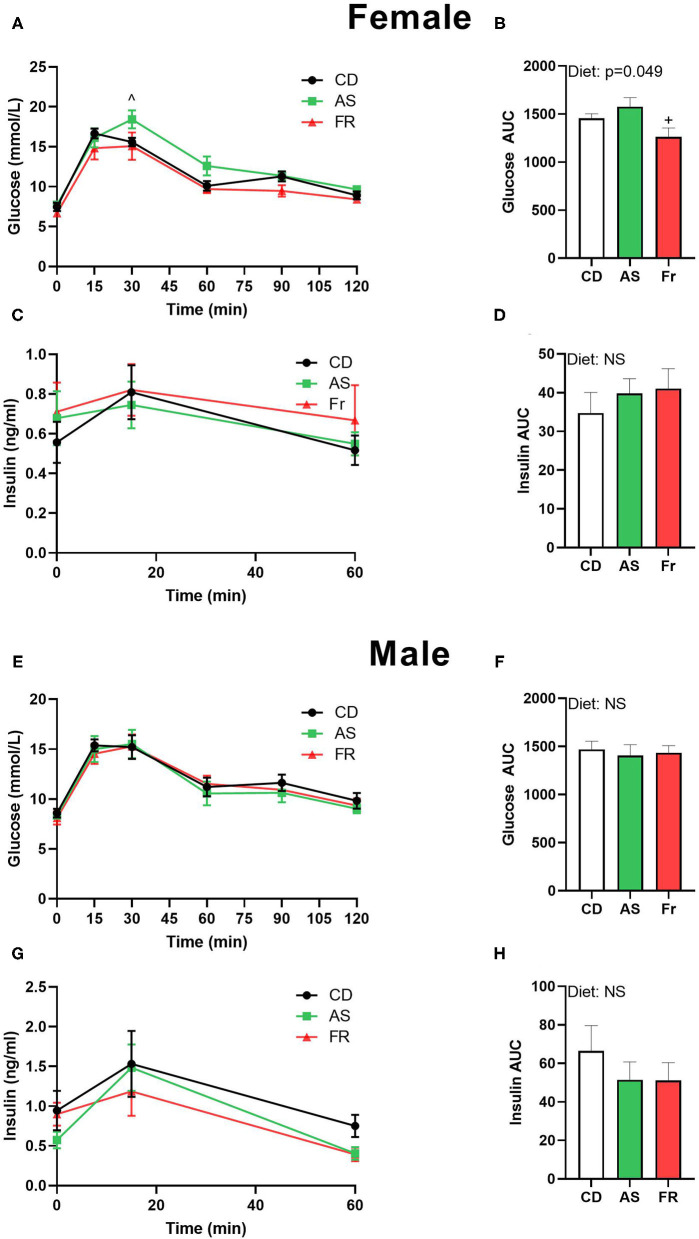
The impact of maternal Ace-K (AS) and fructose (Fr) intake compared to Controls (CD) on glucose homeostasis at 12 weeks of age in female and male C57BL/6 offspring. OGTT (2 g/kg) in female **(A)** and male **(E)** offspring. Area under the OGTT curves in female **(B)** and male **(F)** offspring. Plasma insulin secretion curve at 0, 15, and 60 min post-OGTT in female **(C)** and male **(G)** offspring. Area under the curve in insulin in female **(D)** and male **(H)** offspring. OGTT data were analyzed using repeated measures ANOVA, AUC by ANOVA. Data are expressed as mean ± SEM. +*P* < 0.05 w.r.t AS. ∧*P* < 0.05 w.r.t Fr; *n* = 8 per sex per group.

In female offspring, glucose tolerance in Fr offspring was improved compared to the AS group, which was reflected in a significant difference at the 30 min time point in the OGTT graph and in a decreased OGTT area under the curve (AUC) (Figures 1A,B). There was no difference in insulin concentrations following OGTT in female offspring (Figures 1C,D). In male mice, there was no change across the OGTT or in the AUC between the groups (Figures 1E,F). Similarly, no significant differences were seen in the insulin response curve following the OGTT (Figure 1G) or in the insulin AUC in male mice between groups (Figure 1H).

### Adipocyte Hypertrophy

#### Gonadal Adipose Tissue

**Figure 2:**
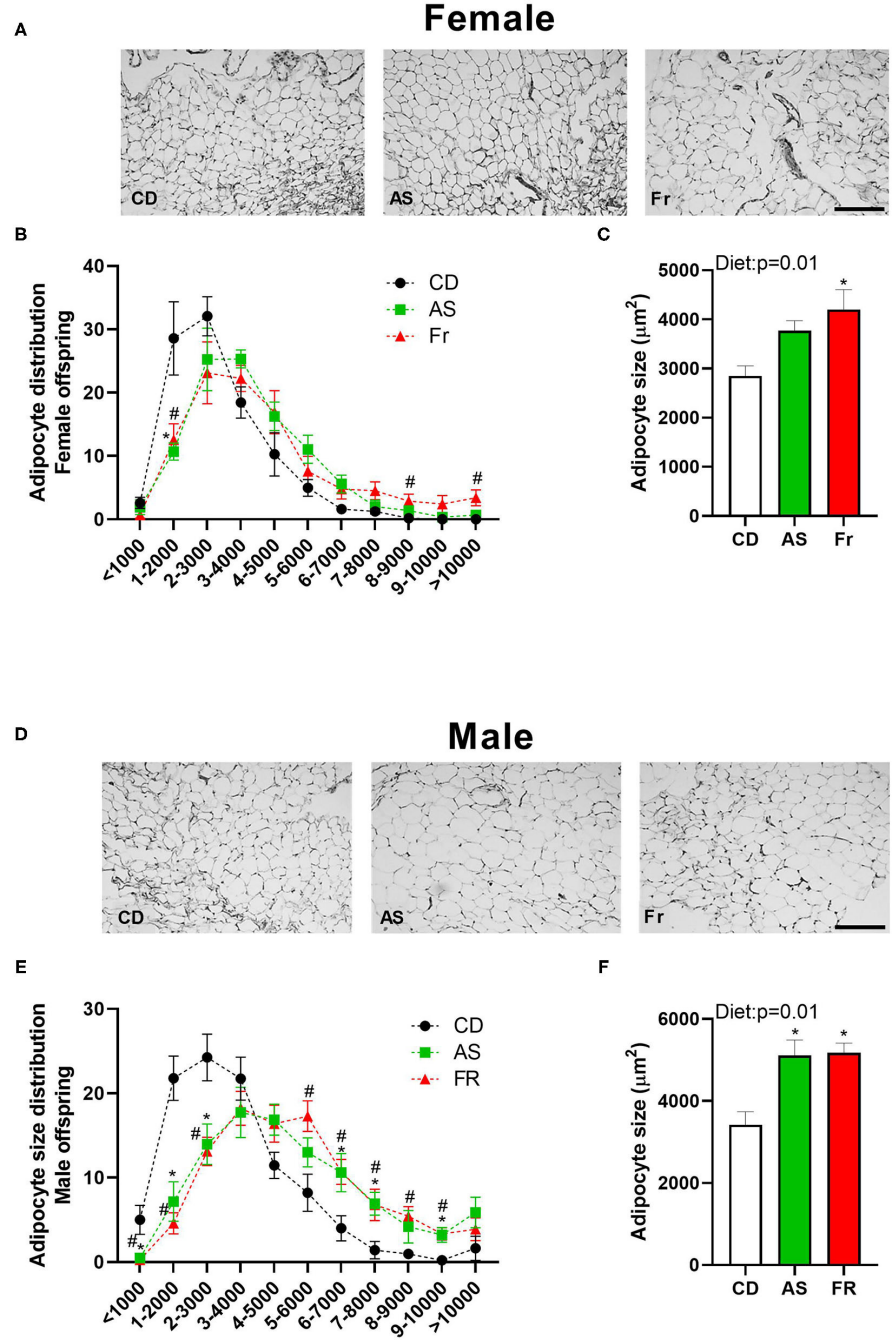
The impact of maternal Ace-K (AS) and fructose (Fr) intake compared to Controls (CD) on adipocyte size in gonadal adipose tissue in female and male C57BL/6 offspring. Representative gonadal adipose tissue sections (hematoxylin and eosin staining) in female **(A)** and male **(D)** offspring. Adipocyte size distribution in female **(B)** and male **(E)** offspring. Adipocyte average size in female **(C)** and male **(F)** offspring. Data were analyzed by one-way ANOVA. Data are expressed as mean ± SEM. *P < 0.05 AS w.r.t CD. #*P* < 0.05 Fr w.r.t CD; *n* = 8 per sex per group.

Average adipocyte size was significantly increased in female offspring in the maternal Fr group as compared to CD group, with AS group trending higher compared to CD group (*p* = 0.067) (Figures 2A,B). Adipocyte distribution varied between the diets in female offspring, with AS and Fr groups reduced at 1–2,000 μm^2^ in size and Fr group increased at 8–9,000 μm^2^ and >10,000 μm^2^ compared to CD group (Figure 2C).

There was an overall increase in the average adipocyte size in both the AS and Fr male offspring groups compared to CD group (Figures 2D,E). In male offspring, CD adipocyte distribution peaked between 1 and 4,000 μm^2^, while AS and Fr distribution peaked at the larger size of 4–7,000 μm^2^. AS and Fr male mice had les adipocytes at <1,000, 1–2,000, and 2–3,000 μm^2^ compared to CD, but more adipocytes in the size range of 6–7,000, 7–8,000, and 9–10,000μm^2^ compared to CD mice. Further, a maternal Fr diet increased the number of adipocytes at 5–6,000 and 8–9,000 μm^2^ compared to CD (Figure 2F).

#### Subcutaneous Adipose Tissue

**Figure 3:**
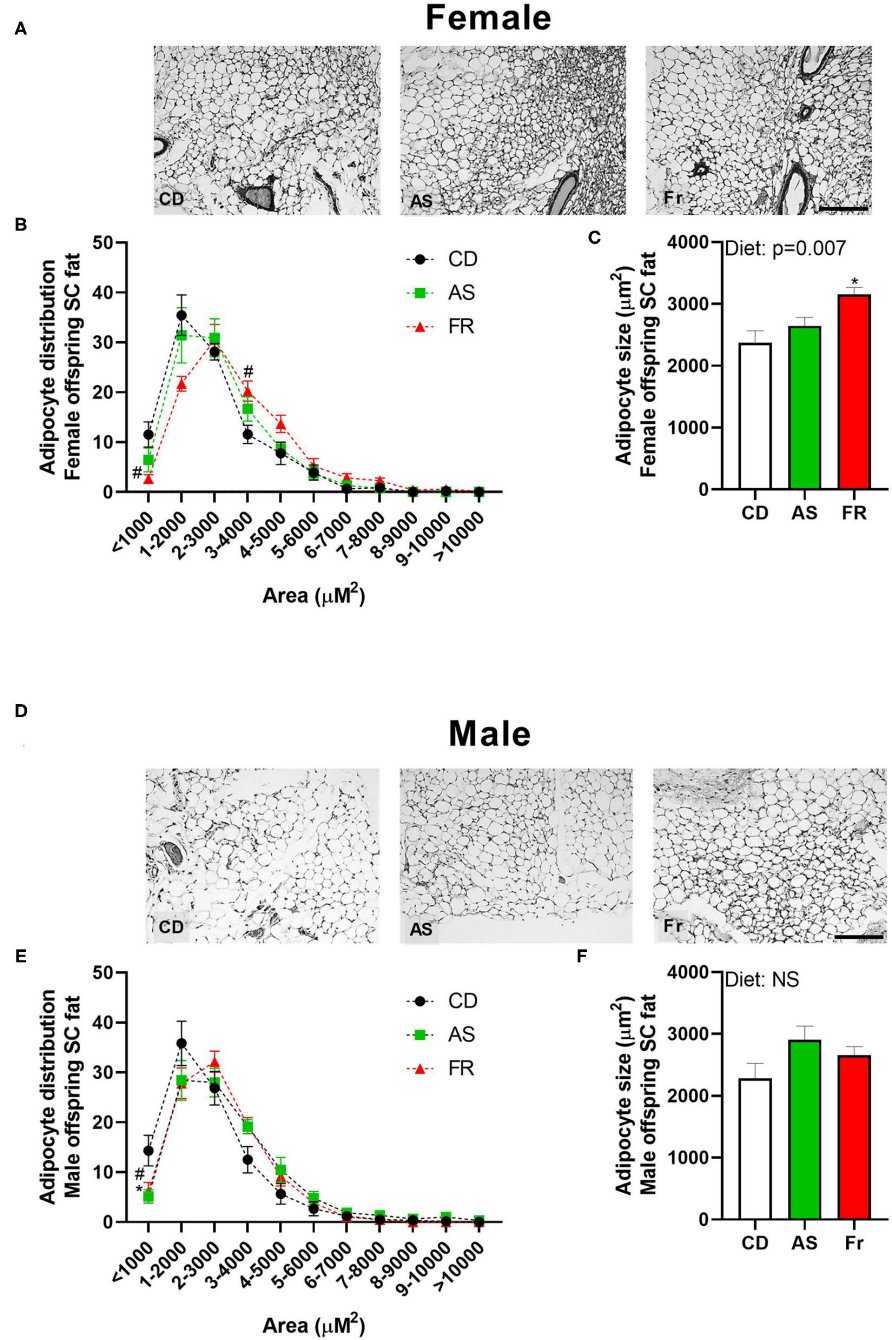
The impact of maternal Ace-K (AS) and fructose (Fr) intake compared to Controls (CD) on adipocyte size in subcutaneous adipose tissue in female and male C57BL/6 offspring. Representative subcutaneous adipose tissue sections (hematoxylin and eosin staining) in female **(A)** and male **(D)** offspring. Adipocyte size distribution in female **(B)** and male **(E)** offspring. Adipocyte average size in female **(C)** and male **(F)** offspring. Data were analyzed by one-way ANOVA. Data are expressed as mean ± SEM. *P < 0.05 AS w.r.t CD. #*P* < 0.05 Fr w.r.t CD; *n* = 8 per sex per group.

There was no overall effect following maternal AS consumption on adipocyte distribution in female offspring. However, Fr female offspring displayed less adipocytes at <1,000 μm^2^ and more adipocytes at 3–4,000 μm^2^ compared to CD (Figures 3A,B), with an overall increase in average adipocyte size (Figure 3C). In male mice, there was a decrease in the number of adipocytes <1,000 μm^2^ in both the AS and Fr groups compared to the CD group (Figures 3D,E), although there was no significant overall difference in average adipocyte size (Figure 3F).

### Adipose Tissue Gene Expression

**Figure 4:**
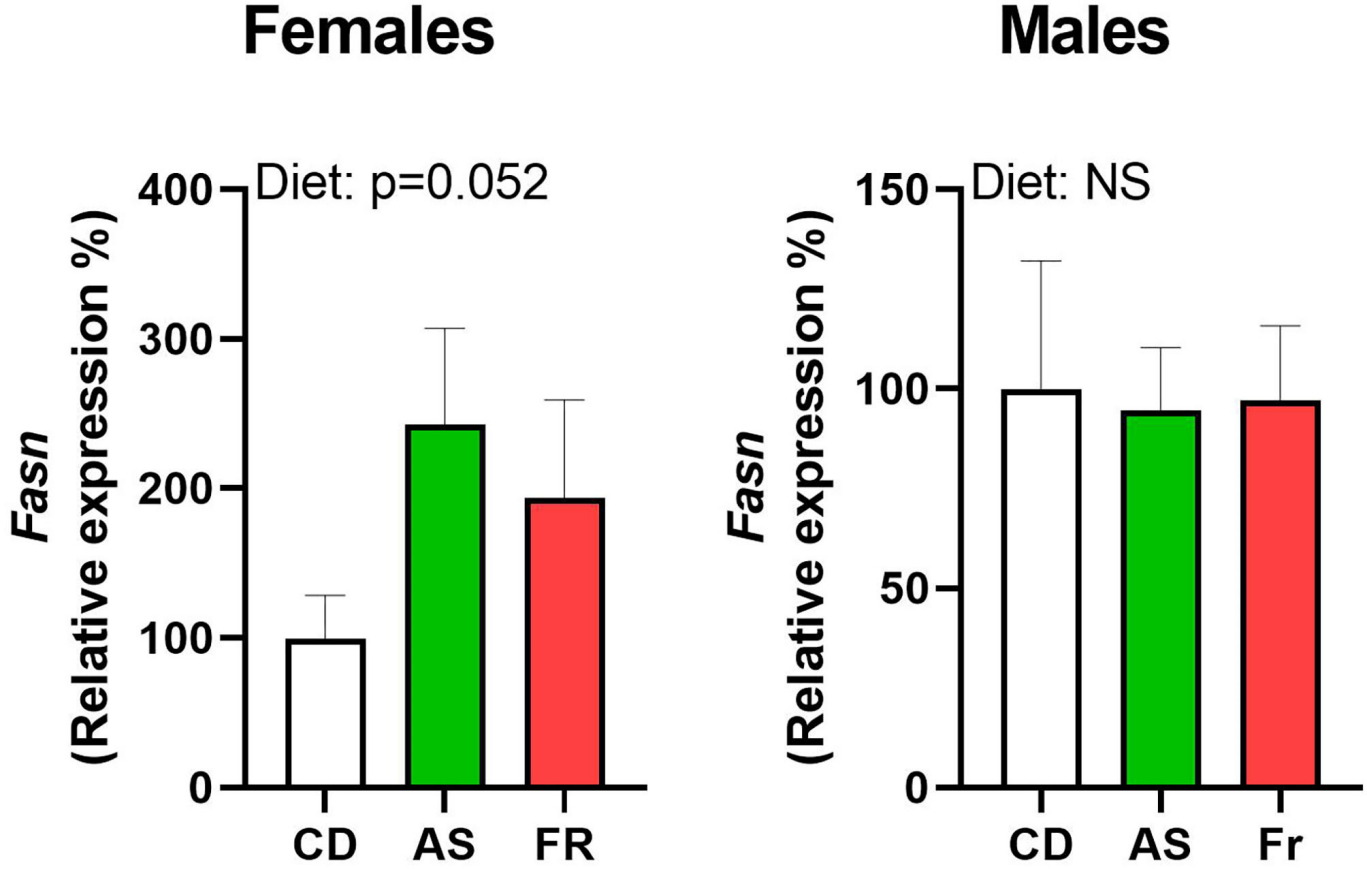
The impact of maternal Ace-K (AS) and fructose (Fr) intake compared to Controls (CD) on *Fasn* gonadal tissue gene expression in female and male C57BL/6 offspring. Data were analyzed using one-way ANOVA. Data are expressed as mean ± SEM. n = 8 per sex per group.

There were no differences in *Fasn* expression in male or female offspring, but there was a strong trend toward an overall maternal dietary effect in females (*p* = 0.052, Figure 4). There were no other differences in gene expression in either male or female offspring across the different maternal dietary groups for Cd68 molecule (*Cd68*), insulin receptor substrate 1 (*Irs1*), forkhead box O1 (*Foxo1*), SRY-box transcription factor 9 (*Sox9*), tumor necrosis factor alpha (*Tnf*α), delta-like non-canonical notch ligand 1 (*Dlk1*), toll-like receptor 4 (*Tlr4*), leptin receptor (*Lepr*), nuclear factor kappa-light-chain-enhancer of activated b cells (*Nfkb*), solute carrier family 2 member 4 (*Slc2a4*), peroxisome proliferator-activated receptor gamma (*Ppar*γ), peroxisome proliferator-activated receptor gamma coactivator 1 (*Ppargc1*), or interleukin-1 (*Il-1*).

### Female Puberty Onset and Estrus Cycle Disruption

**Figure 5:**
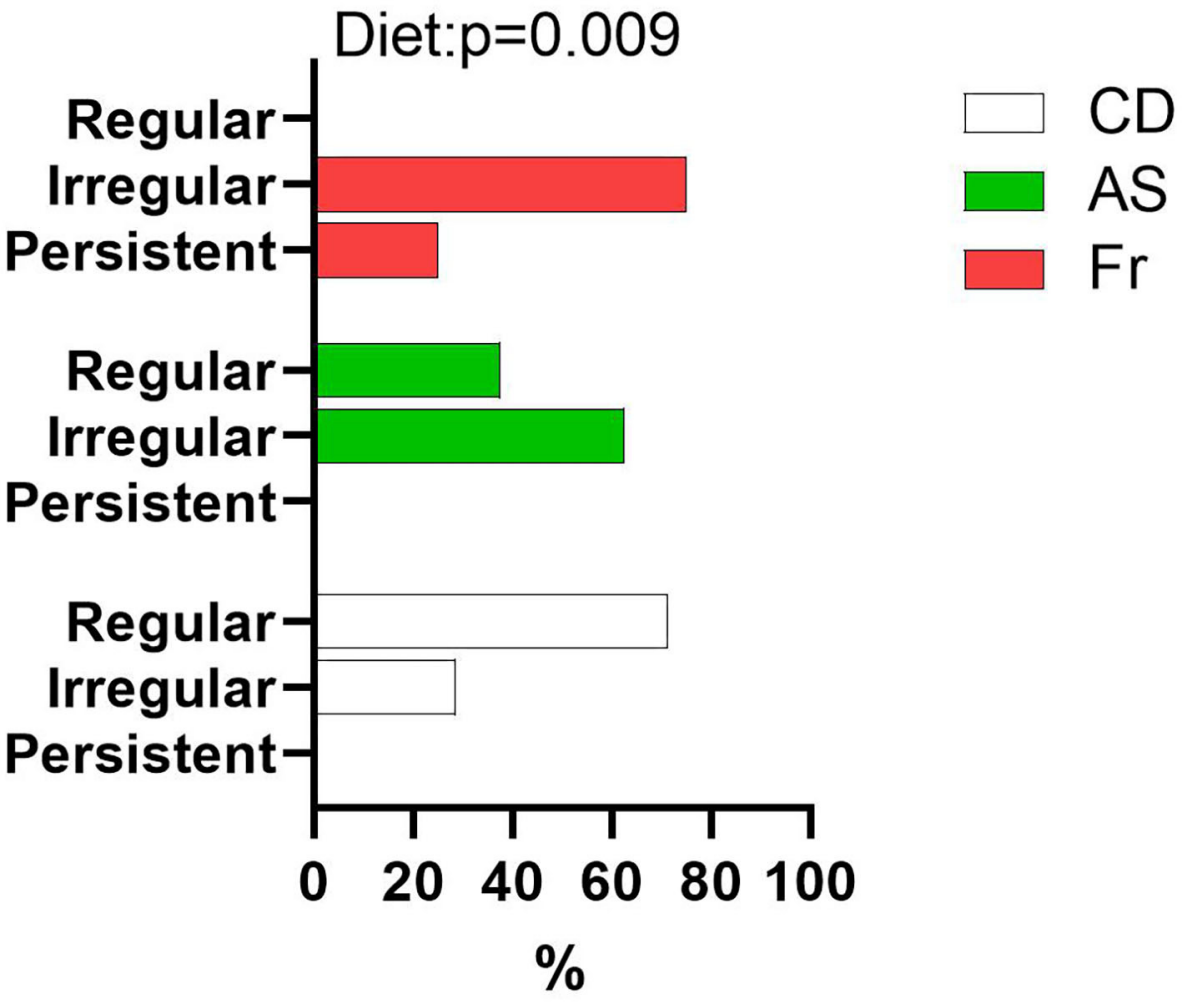
Proportion of female offspring with regular, irregular, or persistent estrus cycles as a percentage of total female offspring within each group. Data were analyzed using nominal logistic regression. *n* = 8 per group.

Maternal diet had no impact on age of puberty onset or body weight at puberty onset in females (Table 1). Female offspring of mothers fed with AS and Fr were more likely to experience irregular estrus cycles compared to CD groups, with those in the Fr group more likely to have persistent estrus compared to all others (Figure 5).

### Ovarian Gene Expression

**Figure 6:**
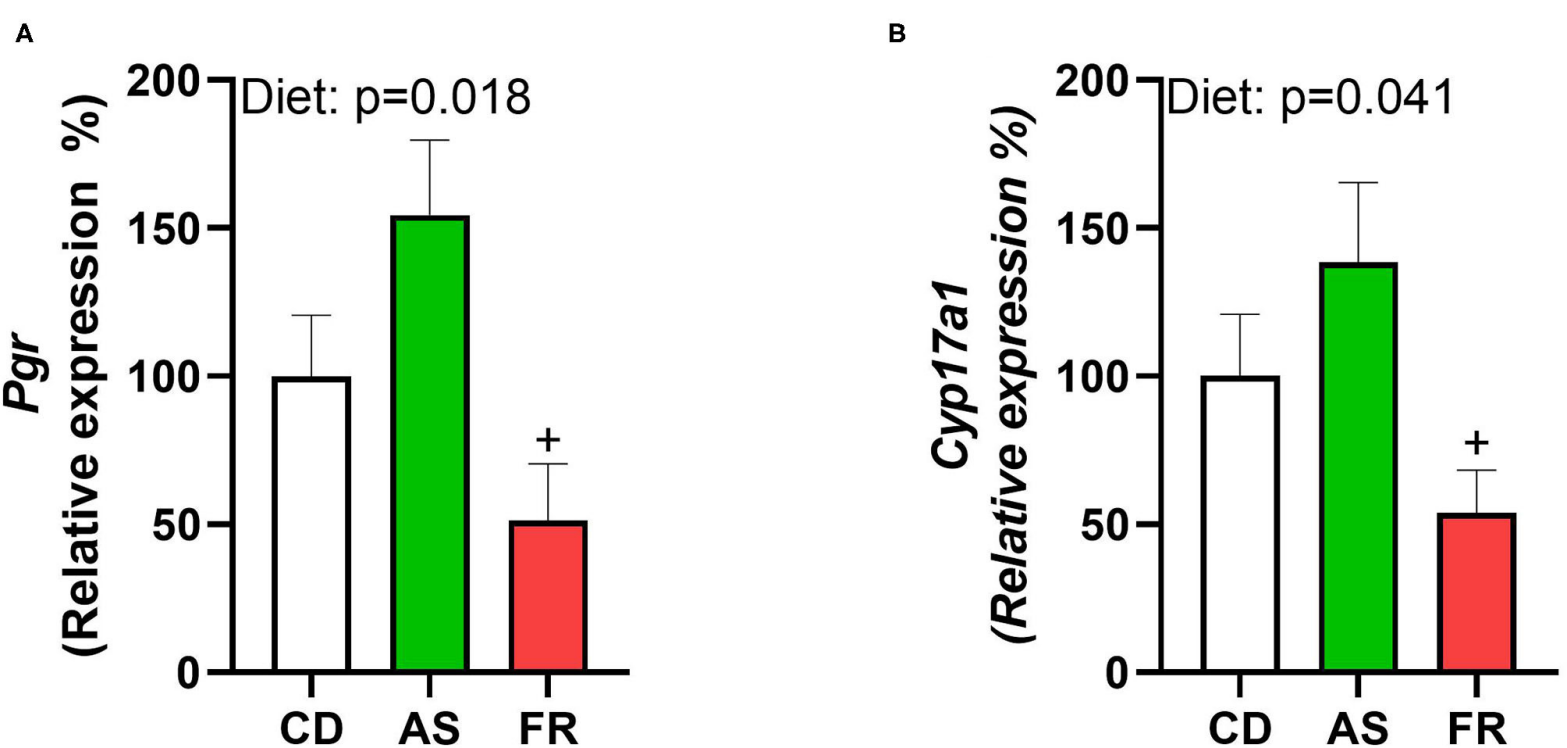
The impact of maternal Ace-K (AS) and fructose (Fr) intake compared to Controls (CD) on ovarian gene expression in female C57BL/6 offspring. **(A)** *Pgr*, **(B)** *Cyp17a1*. Data were analyzed using one-way ANOVA. Data are expressed as mean ± SEM. +P < 0.05 w.r.t AS; *n* = 8 per group.

Maternal consumption of Fr reduced the expression of progesterone receptor (*Pgr*) compared to AS, inducing a significant overall dietary effect (Figure 6A). Cytochrome P450 Family 17 Subfamily A Member 1 (*Cyp17a1*) similarly saw Fr expression reduced compared to AS, with a significant dietary effect (Figure 6B). There were no differences in ovarian gene expression for follicle stimulating hormone receptor (*Fshr*), leptin receptor (*Lepr)*, growth differentiation factor 9 (*Gdf9*), forkhead Box O3 (*Foxo3a*), bone morphogenetic protein 15 (*Bmp-15*), estrogen Receptor 2 (*Esr2*), luteinizing hormone/choriogonadotropin receptor (*Lhcgr*), hydroxysteroid 17-beta dehydrogenase 1 (*Hsd17a1*), or Epiregulin (*Ereg*) across the different maternal dietary groups.

## Discussion

Epidemiological, clinical, and experimental evidence have shown a clear link between an altered early-life environment and increased risk for a range of disorders in offspring in later life. In particular, maternal dietary stressors, such as sugar-sweetened soft drinks high in fructose, contribute to growing rates of metabolic disease in offspring (25). Despite their growing significance in the modern diet, the influence of AS beverages in the maternal diet is less well-known, especially regarding their impact on the metabolic and reproductive health of the offspring. The current study, therefore, utilized a mouse model to examine the effects in offspring following maternal intake of Ace-K, an AS found in most diet soft drinks (22), yet one which is less comprehensively studied. A moderate dose was chosen to better reflect the typical consumption in humans. Despite no change in weight gain, AS and Fr maternal intake increased adipocyte hypertrophy in both male and female mice and altered glucose tolerance in a sex-specific manner. Further, AS and Fr could influence female offspring reproductive systems through alteration of markers associated with ovulation, androgen conversion, and increased risk of irregular estrus cycles.

In a previous study in mice, we reported that AS in the maternal diet reduced male but not female fetal weight, while AS and Fr increased female placental weight compared to CD (15). Neither AS nor Fr maternal consumption altered offspring weight at P2, weaning, or at cull, indicating that the results of this study are independent of body weight. These results may also indicate the possibility of catch-up growth in the male offspring. Few studies have investigated the influence of maternal Ace-K consumption on the health of offspring in mice. However, other studies in mice examining maternal sucrose or sucralose intake have also reported no changes in body weight of offspring (26), while data in the rat in the setting of a maternal Fr diet also reported no differences in birth weight (27). Our results do conflict with studies in human cohorts, which have implicated maternal AS consumption in increased weight at birth and at 7 years of age (12, 14). However, these human cohorts do not distinguish between different AS and may be further influenced by confounding factors and overall dietary patterns that make it difficult to delineate the specific influence of individual sweeteners.

In male offspring, maternal AS consumption reduced plasma glucose concentrations at cull, compared to both the CD and Fr groups, although there was no impact on glucose tolerance following the OGTT. This protective effect was not seen in female offspring at cull, with the reverse being seen, whereby the OGTT revealed that glucose intolerance was increased in female offspring of mothers fed with AS compared to the Fr group. Several studies link maternal fructose diets to increased obesity and IR in offspring (28). In many studies, different concentrations of Fr or AS may cause differences in outcome, with supraphysiological concentrations likely to exacerbate the effects. Further, while Saad et al. used a similar amount of Fr in their diets (10–15% kcal/d compared to 20% kcal/d in this study), their mice were 1 year old at the time of cull (28) compared to 14 weeks in the current study. Aging is often associated with reduced efficacy of adipocytes (29), and may therefore further influence the negative effects seen in these mice. It would therefore be of interest to extend this current experiment and follow mice through to an older age to determine if obesity or other metabolic effects are exacerbated with age. Furthermore, there is evidence that maternal diet-induced developmental programming primes offspring metabolic dysfunction, and therefore this requires a “second hit” such as a post-natal high-fat diet (HFD) to demonstrate the negative impact of *in utero* exposures and reveal any latent disease (30).

With this in mind, we assessed adipocyte morphology. Despite no difference in weight gain, maternal Fr and AS induced adipocyte hypertrophy in gonadal adipose tissue in male offspring. In females, this was only seen in response to Fr, though the AS group did display a strong trend toward increased adipocyte size when compared to CD group. In both male and female offspring, the distribution of adipocytes was skewed toward larger sizes following both Fr and AS exposure. Adipocyte hypertrophy, even in the absence of obesity, is a common predictor of adipose metabolic dysfunction, dyslipidemia, IR, and T2DM. It is linked to increased recruitment of macrophages, and other pro-inflammatory adipokines (31, 32). This is especially true in visceral adipose tissue depots, which are more metabolically active than subcutaneous adipose tissue (33), though subcutaneous adipose tissue can still contribute to metabolic dysfunction when impaired (34). Hypertrophy of subcutaneous adipocytes was seen in female offspring of Fr-fed mothers, but not male offspring, indicating that female offspring might have a more systemic influence from the mother's diet on their adipose tissue morphology than male offspring.

To further investigate the influence of maternal AS and Fr intake on offspring metabolic health, a panel of genes associated with adipogenesis, inflammation, and specific metabolic pathways were examined. Female, but not male offspring, displayed a strong trend toward increased expression of *Fasn* following maternal AS and Fr intake. *Fasn* is involved in lipogenesis and associated with reduced insulin sensitivity and obesity, with expression increased by insulin within human adipocytes (35). This trend in female offspring could be indicative of an increased risk to insulin sensitivity and later-life fat accumulation (35). Despite evident adipocyte hypertrophy, male offspring exhibited no differences in the expression of genes examined in this study, indicating that male offspring may not suffer from the same negative perturbations in these investigated pathways. As previously mentioned, one theory associated with the role that maternal nutrition plays in the later health of offspring is that it primes offspring for later metabolic dysfunction that may become apparent after a secondary nutritional or environmental derangement, such as an HFD. Further, AS has been found in both amniotic fluid and breast milk (16, 17), with suggestions that this may alter the gut microbiome of the offspring and/or change their taste preference (18). A switch toward a sweet preference could result in the offspring favoring high-sugar foods in later life, a diet associated with increased obesity, and therefore inducing this secondary nutritional challenge. In our study, offspring were maintained on water and standard control diet until cull, therefore we were unable to test whether a second insult would exacerbate any potential metabolic disease. However, if female offspring from the AS group are more susceptible to fat accumulation, later changes in diet could potentially produce these latent effects.

While the influence of maternal nutrition on the metabolic health of offspring has been frequently investigated, less is known about the potential impact of maternal diets that include AS and Fr on the reproductive health of offspring. While puberty onset was found to be unchanged between female offspring, irregular estrus cycles were increased in female offspring of AS and Fr mothers. Maternal HFD exposure has been shown to induce increased estrus cycle irregularity in female mice offspring (36), confirming the ability of altered maternal nutrition to influence aspects of reproductive health of offspring. Leptin and insulin deficiency have both been associated with alterations to female reproductive capacity; however as there was no change in either, it is likely that another mechanism is influencing the estrus cyclicity of female offspring. We assessed ovarian gene expression to further investigate estrus irregularities. *Pgr* and *Cyp17A1* gene expressions were both reduced following maternal Fr consumption compared to AS. PGR is expressed in granulosa cells of preovulatory follicles and mediates the effects of progesterone, which is involved in the regulation of ovarian function and ovulation (37). Female offspring of rat dams fed with fructose also displayed reductions in ovarian expression of *Pgr*, with maternal fructose consumption implicated in the impairment of estradiol homeostasis in female offspring (38). Cyp17A1 is involved in the conversion of progesterone to 17α-hydroxypregnenolone and then to androgens. High expression of CYP17A1 has been implicated in increased androgen concentrations commonly found in women with PCOS (37, 39), as has irregular menstrual cycles (40). Like PGR, CYP17A1 is regulated by luteinizing hormone (LH). It is possible that offspring of AS mothers have higher LH concentrations, which cause this increase in ovarian gene expression, while Fr may influence estradiol synthesis. It would be worth further exploring reproductive function and related steroid hormones within the female offspring.

Fructose increased plasma testosterone in male, but not female, offspring compared to both CD and AS groups. This agrees with a previous study where peripubertal male Wistar rats were fed Fr for 30 days, resulting in increased plasma testosterone concentrations and impaired testicular and epididymal development (41). This study was particularly interested in the reproductive health of female offspring, given the propensity to influence the next generation and the subjectivity around assessing the puberty onset of the male offspring. However, given this association between increased testosterone and modified male reproductive systems, and increasing interest in the role of paternal factors in developmental programming (42), further studies examining the influence of maternal AS and Fr intake on male reproductive health are warranted.

This study utilized a model of altered maternal nutrition in the mouse, whereby AS intake was equivalent to a standard can of diet soda a day. While many studies utilize supraphysiological concentrations of AS and Fr to induce effects, we have been able to show that lower concentrations can exert an influence on offspring following maternal consumption. Undertaking research across other ranges of AS and Fr intakes could help further elucidate the impact of diet soda consumption on the health of offspring. It would also be worthwhile undertaking a comparative study across a broader range of AS, including natural-based sweeteners such as Stevia. Further, our study ceased at 14 weeks of age. At times, the effects of developmental programming seen in offspring studies are subtle, as larger disturbances to metabolic health can increase the chance of mortality. A second insult in later life may be required to exacerbate potential disease. It would therefore be interesting to continue this study paired with a HFD in later life to examine possible interactions between maternal exposures and later susceptibility of offspring to diet-induced obesity. However, the results of the present study do indicate an impact, albeit subtle, of early life exposure to AS and Fr on later health outcomes. It may also be of interest to investigate the same parameters in the setting of maternal obesity and/or GDM, as this may exacerbate metabolic dysfunction in the offspring. This study did not investigate the gut microbiome of offspring. Given the recent insight into the influence of the gut microbiome on systemic health and the ability of AS to influence the gut microbiome, it would be a worthwhile avenue to investigate (5, 43). Further, due to limited samples, the analysis of plasma proteins and steroid hormones could not be performed. These might have given further insights into the metabolic and reproductive health of the animals. Nonetheless, PCR analysis enabled valuable analysis of a number of metabolic and reproductive markers.

In summary, a maternal diet of AS and Fr over pregnancy and lactation induced subtle metabolic and reproductive effects in male and female offspring, which were often sex-specific. Female offspring were more susceptible to glucose intolerance following a maternal AS diet. Maternal Fr and AS intake negatively impacted estrus cyclicity, and potentially impaired ovarian steroid hormone synthesis, although more investigation is required to fully elucidate these effects. Conversely, male offspring exhibited reduced baseline glucose concentrations in the setting of a maternal AS diet, and no alterations to metabolic adipose markers, thereby emphasizing the importance of undertaking studies in both males and females. Finally, the demonstrated adipocyte hypertrophy may indicate the potential for the development of later metabolic dysfunction should the offspring be exposed to another negative environmental influence, such as a postnatal HFD. As such, the present study adds to the experimental evidence to date suggesting that AS may not be beneficial alternatives to sugar-sweetened products consumed during pregnancy and early infancy.

## Data Availability Statement

The authors acknowledge that the data presented in this study must be deposited and made publicly available in an acceptable repository, prior to publication. Frontiers cannot accept a manuscript that does not adhere to our open data policies.

## Ethics Statement

The animal study was reviewed and approved by Animal Ethics Committee at the University of Auckland (Approval number 001846) in accordance with the New Zealand Animal Welfare Act, 1999.

## Author Contributions

Conceptualization was done by CR. Methodology was carried out by CR, PB-C, and MV. Data collection was done by CR, AS, JR, JM-J, and PB-C. Data analysis was performed by CR, PB-C, and JR. Writing—original draft preparation was done by PB-C. Writing—review and editing was done by CR, PB-C, and MV. Supervision was carried out by CR and MV. Project administration, was done by CR and MV. Funding acquisition was taken care of by CR. All authors have read and agreed to the published version of the manuscript.

## Funding

This work was supported by funding from the Faculty Research Development Fund (#3710414) at the University of Auckland and The University of Auckland Foundation (#3721401). PB-C was supported by a Maori Health Research PhD Scholarship from the Health Research Council of New Zealand (#3714791). MV is supported by a James Cook Research Fellowship from the Royal Society of New Zealand. CR is supported by an Ad Astra fellowship from University College Dublin.

## Conflict of Interest

The authors declare that the research was conducted in the absence of any commercial or financial relationships that could be construed as a potential conflict of interest.

## Publisher's Note

All claims expressed in this article are solely those of the authors and do not necessarily represent those of their affiliated organizations, or those of the publisher, the editors and the reviewers. Any product that may be evaluated in this article, or claim that may be made by its manufacturer, is not guaranteed or endorsed by the publisher.
